# BNLoop-GAN: a multi-loop generative adversarial model on brain network learning to classify Alzheimer’s disease

**DOI:** 10.3389/fnins.2023.1202382

**Published:** 2023-06-23

**Authors:** Yu Cao, Hongzhi Kuai, Peipeng Liang, Jeng-Shyang Pan, Jianzhuo Yan, Ning Zhong

**Affiliations:** ^1^Faculty of Information Technology, Beijing University of Technology, Beijing, China; ^2^Beijing International Collaboration Base on Brain Informatics and Wisdom Services, Beijing, China; ^3^Faculty of Engineering, Maebashi Institute of Technology, Maebashi, Gunma, Japan; ^4^School of Psychology and Beijing Key Laboratory of Learning and Cognition, Capital Normal University, Beijing, China; ^5^College of Computer Science and Engineering, Shandong University of Science and Technology, Qingdao, China

**Keywords:** BNLoop-GAN model, multiple-loop-learning, evidence combination-fusion computing, magnetic resonance imaging, brain network analysis, Alzheimer’s disease

## Abstract

Recent advancements in AI, big data analytics, and magnetic resonance imaging (MRI) have revolutionized the study of brain diseases such as Alzheimer’s Disease (AD). However, most AI models used for neuroimaging classification tasks have limitations in their learning strategies, that is batch training without the incremental learning capability. To address such limitations, the systematic Brain Informatics methodology is reconsidered to realize evidence combination and fusion computing with multi-modal neuroimaging data through continuous learning. Specifically, we introduce the BNLoop-GAN (Loop-based Generative Adversarial Network for Brain Network) model, utilizing multiple techniques such as conditional generation, patch-based discrimination, and Wasserstein gradient penalty to learn the implicit distribution of brain networks. Moreover, a multiple-loop-learning algorithm is developed to combine evidence with better sample contribution ranking during training processes. The effectiveness of our approach is demonstrated through a case study on the classification of individuals with AD and healthy control groups using various experimental design strategies and multi-modal brain networks. The BNLoop-GAN model with multi-modal brain networks and multiple-loop-learning can improve classification performance.

## 1. Introduction

The rapid advancement of AI and big data technologies have revolutionized the field of brain investigation, providing new insights into its workings and potential applications. However, medical research on the brain presents more significant challenges as it involves navigating the complex interplay of biological, psychological, and environmental factors. In response to this, the Brain Informatics (Zhong et al., 2011) methodology has been proposed to study the mechanisms underlying the human information processing system with big data (Zhong et al., 2005). As the core part of Brain Informatics, a series of “evidence combination-fusion computing (ECFC)” methods (Kuai et al., 2022) are developed to promote fundamental and translational studies of the brain, encouraging to handle multi-source brain big data continuously during learning and validating phases of models and systems. The continuous learning enables the more effective utilization of existing information and experiences learned by previous data, which are different from the current most machine learning algorithms.

Alzheimer’s Disease (AD), as a neurodegenerative disease that occurs frequently in the elderly, has become a severe threat to the health, with clinical manifestations of cognitive decline, accompanied by other physiological or mental disorders (Citron, 2010; Ferrari and Sorbi, 2021). In recent years, Magnetic Resonance Imaging (MRI) technology has emerged as a valuable tool in diagnosing AD due to its non-radiative, non-invasive, and non-harmful characteristics. In particular, it offers high tissue resolution and can be utilized for imaging with a variety of parameters (Jin et al., 2020; Cao et al., 2022). However, single modality-based investigations may not provide sufficient information to identify complex diseases. The multi-modal MRI techniques, such as diffusion MRI (dMRI) and functional MRI (fMRI), can provide a holistic view to observe changes in brain structure and function of AD (Cuingnet et al., 2011; Zhang et al., 2020). In the context, considering the advantages of complementary information, multi-modal analyses corresponding to both structural and functional characteristics have a great boom simultaneously (Poldrack and Farah, 2015). Furthermore, the brain network analysis has been widely employed in the diagnosis of brain diseases, which can provide valuable insights into the connected mechanisms between different brain regions (Lama and Kwon, 2021). For instance, dMRI (Soares et al., 2013) has been utilized to construct structural connectivity to measure the connections of nerve fiber bundles in white matter, while resting-state functional MRI (rsfMRI) (Sheline and Raichle, 2013; Soares et al., 2016) has been used to construct functional connectivity to detect the functional activity of the brain.

Recent advancements in AI, particularly Generative Adversarial Networks (GANs), have demonstrated great potential in analyzing complex brain data. GANs are capable of learning and generating new data samples that resemble the input data (Fahimi et al., 2020). In the context of AD, GANs can be trained on large datasets of brain images to learn patterns associated with the disease, helping in the diagnosis of AD by identifying subtle changes in brain structure or function. However, the current limitations of most AI models in neuroimaging classification tasks lead to underutilization of existing information and insufficient processing of unbalanced data. The primary challenge lies in the strategy of randomly selecting data for training at once, which ignores the potential benefits of utilizing data systematically and continuously.

Confronted with the complexity of these brain science problems, the Brain Informatics methodology provides a systematic perspective to understand the principles and mechanisms of human information processing related to high-order cognition functions cognitive functions (such as reasoning, calculation and problem solving) (Yang et al., 2009), as well as the development of new technologies for analyzing the biological characteristics and clinical applications on brain diseases. In the context of Brain Informatics, multi-modal and multi-scale brain data are analyzed systematically by considering different distributions of samples, so as to personalized applications. For example, the Data-Brain driven general intelligence model (Kuai and Zhong, 2020) is proposed to realize systematic brain computing in terms of the diversities of brain data from the experimental perspective. In particular, an iterated and evolved computing cycle was designed to continuously evidence combination and fusion computing.

In this paper, we propose the BNLoop-GAN model, which couples the Loop-based Generative Adversarial Network with the ECFC method for multiple loop brain network learning. The main contributions of this study can be summarized as follows: (1) an enhanced-GAN model is developed, utilizing techniques such as conditional generation, patch-based discrimination, and Wasserstein gradient penalty to learn the implicit distribution of brain networks; (2) a multiple-loop-learning algorithm is introduced, which combines evidence with better sample contribution ranking during continuous training phases; (3) the BNLoop-GAN model is applied to a case study of AD classification, where single-modal and multi-modal brain networks are computed iteratively to improve classification performance.

The rest of this paper is organized as follows: Section 2 provides a review of related works on brain networks, GANs, and AD. Section 3 introduces the overall framework of the BNLoop-GAN model for classification tasks, which comprises an enhanced GAN model and a multiple-loop-learning algorithm. Section 4 describes the experimental settings, data preparation, brain network construction, and performance evaluation. Section 5 presents results and discusses different scenarios on single-modal and multi-modal brain networks. Finally, Section 6 gives a conclusion and outlines future work.

## 2. Related work

Recently, AI models have gained widespread popularity in image generation, image super-resolution and other requirements based on their generative capabilities of addition, deletion, and modification. In the medical field, GAN models have been widely applied to diagnosis of AD. For instance, Yu et al. (2022) proposed a Multidirectional Perception GAN that uses a multidirectional mapping mechanism to learn morphological features for classifying AD severity at different stages. Yu et al. (2021) also proposed a three-player cooperative game-based framework with the high-order pooling scheme, namely tensorizing GAN, which is used to learn the structural information of MRI to assess mild cognitive impairment and AD. Moreover, a condition GAN (cGAN) model (Jung et al., 2023) is proposed to generate high-quality 3D MR brain images at different stages of AD, which integrates an additional module to ensure smooth and realistic transitions in 3D space, and uses an adaptive identity loss to preserve patient identification features. Ji et al. (2021) proposed a framework utilizing recurrent GANs for estimating effective connectivity from rsfMRI data, revealing potential differences in neural influence and information flow between AD and healthy control (HC) groups.

Given the complexity of AD, many studies have paid attention to use GAN models for multi-modal neuroimaging analysis. Pan et al. (2021) developed a Decoupling GAN to detect abnormal neural circuits for AD, which decomposes a brain network into two parts and utilizes an analytic module associated with the hyperedge neurons algorithm. The proposed model can extract complementary topology information between rsfMRI and diffusion tensor imaging (DTI) to detect abnormal neural circuits at different stages of AD. Moreover, a cross-modal transformer GAN (Pan and Wang, 2022) has been introduced, which employs a bi-attention mechanism to merge rsfMRI and DTI data effectively, facilitating the identification of AD-associated brain connectivity and enhancing the accuracy of classification. Zuo et al. (2021) developed a multi-modal representation learning and adversarial hypergraph fusion framework using complete trimodal images (MRI, DTI and rsfMRI) to address the limitation of data distribution inconsistency in AD diagnosis. Zuo et al. (2021) also developed a prior guided adversarial representation learning and hypergraph perceptual network, which can evaluate the changing characteristics of brain connectivity at different stages of AD.

With the progress of brain connectivity, brain network analyses break a new ground in the study of AD. Cui et al. (2018) developed a minimum spanning tree method to construct the brain functional network, and extracted the topological features of the brain network. They used the support vector machine to compare AD and HC groups. Islam and Zhang (2018) proposed a deep convolutional neural network to learn features from a small and imbalanced dataset of structural MRI, which can identify and classify AD at different stages. Ye et al. (2019) selected DTI from 161 participants and used multivariate distance matrix regression (MDMR) analysis to detect structural abnormalities of brain networks during the development of AD disease. On the basis of the seed regions selected by MDMR analysis, supervised learning was applied to evaluate the predictive performance of AD. Furthermore, Zhang et al. (2022) proposed a multi-graph convolutional network based on GAN, which can learn the complex relationship between individual brain structural and functional networks automatically. Lei et al. (2021) proposed an automatic weighted centralized multi-task learning framework, in which multi-task learning is applied to identify features integration of structural and functional connectivity, for providing new insights into early AD detection.

Considering the complexity and systematization of brain computing in current big data era, the loop-based strategy is adopted to perform continuous learning inspired by Brain Informatics methodology. For example, Kuai et al. (2021) proposed the ECFC method to analyze multi-task fMRI data from different sources through merging systematic experimental design with evidence type reasoning. The uncertainty is analyzed and inferred to provide finer interpretations from both cognitive functions and brain regions. Furthermore, the similar strategy is adopted to decode the hidden relationship between connectivity abnormalities and brain disorders as well (Kuai et al., 2021). However, these methods only concern with fMRI at a single modal. In this paper, we extend the loop-inspired method from single modal to multiple modals, and from cognitive functions to brain diseases. In the next section, we will introduce how to realize a GAN-driven multiple-loop-learning to carry out systematic brain big data computing.

## 3. Methods

### 3.1. Overview

**Figure 1:**
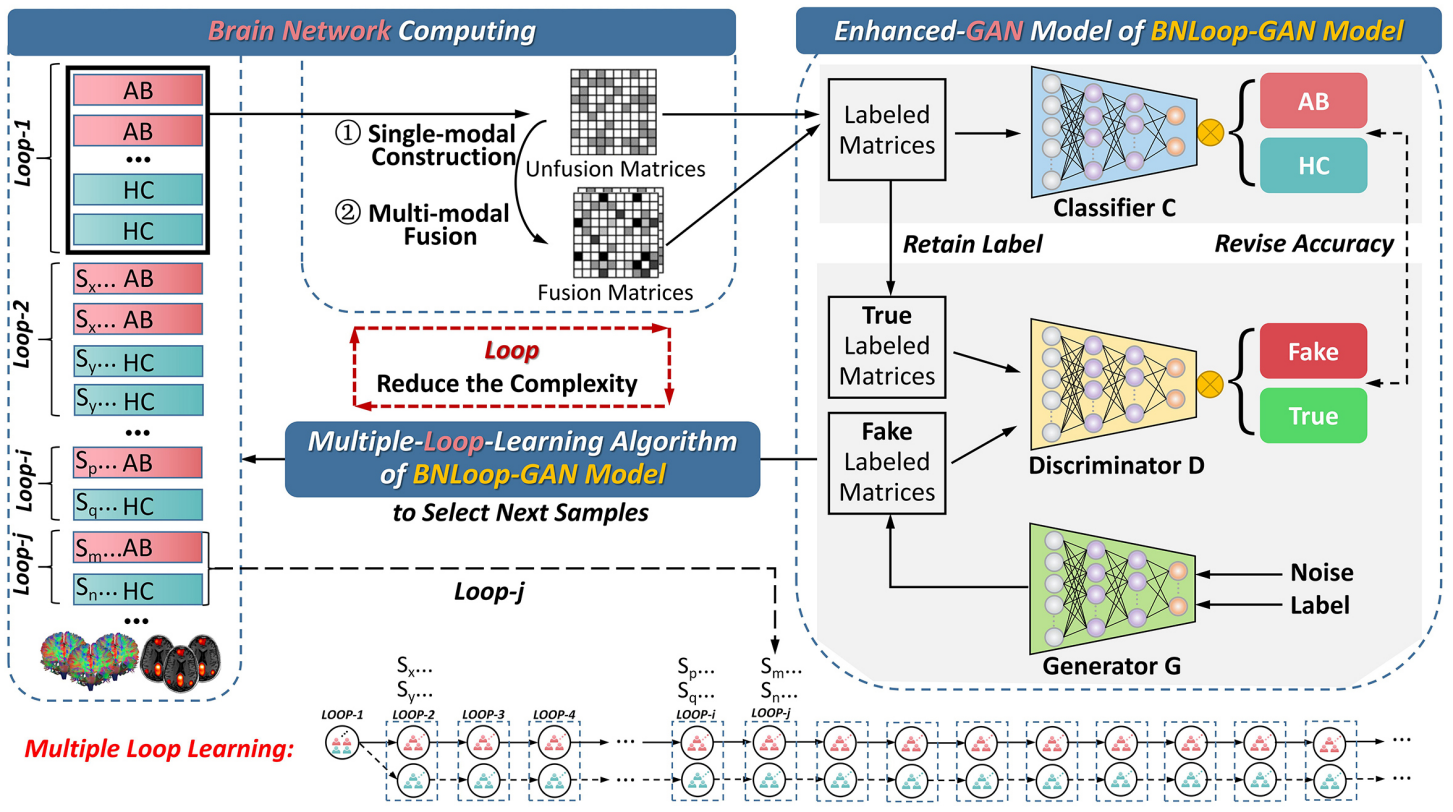
The overall framework for the classification of brain networks. AB, abnormal groups; HC, healthy control groups.

In this section, we introduce the overall framework for addressing the classification task of brain networks between abnormal and HC groups. The framework consists of three main components, which is illustrated in Figure 1. The first component is the brain network computing component, by which both structural and functional brain networks are obtained by analyzing multi-modal brain images. The second and third components are the enhanced-GAN model and the multiple-loop-learning algorithm, respectively, both of which constitute the Loop-based Generative Adversarial Network model for Brain Network (BNLoop-GAN). Before brain networks are learned, some preprocessing steps are required, including: the multi-modal MRI data are processed, such as denoising, calibration, correction; and then brain networks are constructed, such as brain region selection, region segmentation, time series extraction and connectivity measures. Afterwards, the constructed brain networks are recognized by the BNLoop-GAN model with Classifiers to realize classification tasks. During this process, a multiple-loop-learning algorithm is used to select the small batch of samples from the whole training set step by step. The selected samples have an easier-to-learn probability distribution, which can reduce the complexity of model training. Each round of training processes is considered as a loop ( $Loop-i,Loop-j|i,j\in N^{+}$ ), in which the same number of samples from abnormal and HC groups are selected ( $S_{x,y,p,q,m,n}|x,y,p,q,m,n\in N^{+}$ ), where x,p,m represents the number of abnormal groups; y,q,n represents the number of HC groups; x=y,p=q,m=n. More specifically, a multiple-loop-learning algorithm is developed, depending on the training loop from previous iterations.

### 3.2. The enhanced-GAN model

**Figure 2:**
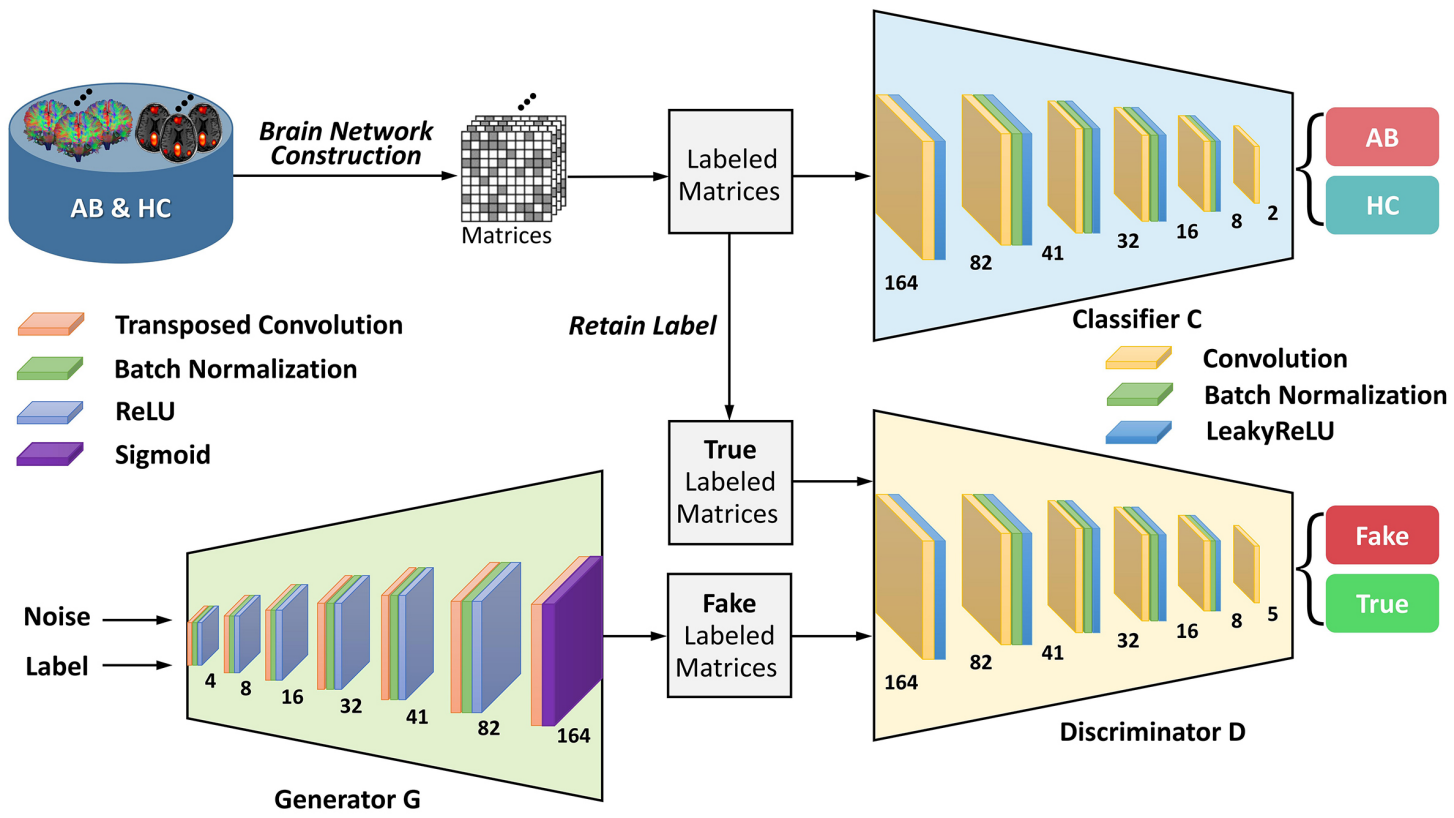
The architecture of the enhanced-GAN model. AB, abnormal groups; HC, healthy control groups.

To provide greater clarity on the enhanced-GAN model within the BNLoop-GAN model, illustrated in Figure 2, we present further details on its constituent components: a generator, a discriminator, and a classifier. The generator is structured with transposed convolutional layers, batch normalization, and activation functions such as ReLU and Sigmoid. Similarly, the discriminator and classifier share the similar structure, both of which are composed of convolutional layers, batch normalization, and LeakyReLU activation functions.

To provide support for the generation of matrices with specified attributes in the subsequent multiple-loop-learning algorithm, the enhanced-GAN model incorporates conditional information into both the generator and discriminator. This is accomplished through the use of a conditional GAN (cGAN) (Mirza and Osindero, 2014) architecture, which enables the model to better comprehend the contextual information of the generation task.

Patch-based processing is commonly utilized in computer vision tasks, including image analysis and object recognition, because it allows for local analysis of image features. This approach can be especially useful when dealing with complex symmetric matrices, as it enables the network to focus on smaller, more manageable sections of the input at a time. The idea of PatchGAN (Isola et al., 2017) is combined here to map the input to N∗N patches. These patches are designed to process matrices in a “patch-wise” manner, meaning that they divide the input image into small overlapping patches and process each patch individually. By learning the brain regions using block features, it is possible to gain a deeper understanding of how the network is processing and interpreting the input matrix at a local level. This information can be useful for identifying patterns or features within the brain regions that are important for the network’s decision-making process and for improving the performance of the network on the discrimination task.

Wasserstein GAN with gradient penalty (WGAN-GP) (Gulrajani et al., 2017) is added to address the problems of traditional GANs (Goodfellow et al., 2014), such as mode collapse and training instability. The core concept of WGAN-GP is to use Wasserstein distance to measure the difference between the generated and real data distributions and to enforce the Lipschitz continuity of the network through gradient penalty. Compared to traditional Wasserstein GAN (Arjovsky et al., 2017), WGAN-GP has the advantage of providing more stable training performance and producing better sample quality of brain networks. The definition of Wasserstein distance is shown as follows:

(1) $$W\left(P_{r},P_{g}\right)=\inf_{\gamma ∼\prod \left(p_{r},p_{g}\right)}E_{\left(x,y\right)∼\gamma }\left[\left\|x-y\right\|\right]$$

where $P_{r}$ is the real distribution and $P_{g}$ is the model distribution implicitly defined by the generator; $\prod \left(p_{r},p_{g}\right)\;$ denotes the set of all joint distributions γ(x,y) whose marginal distributions are $P_{r}$ and $P_{g}$ respectively; $E_{\left(x,y\right)∼\gamma }\left[\left\|x-y\right\|\right]$ is the mathematical expectation of distance $\left\|x-y\right\|$ ; and $\inf\left\{\cdot \right\}$ is the lower bound of set.

In order to solve the mode collapse and improve the convergence speed of traditional GANs, the gradient penalty is added to the discriminator loss function, and the generated samples are constrained by Lipschitz. The discriminator loss function is:

(2) $$\begin{array}{l} L_{D}=E_{\tilde{x}\sim P_{g}}\left[D\left(\tilde{x}\right)\right]-E_{x\sim P_{r}}\left[D\left(x\right)\right]\\ +\;\lambda E_{\hat{x}\sim P_{\bar{x}}}\left[{\left({\left\|\nabla_{\hat{x}}D\left(\hat{x}\right)\right\|}_{2}-1\right)}^{2}\right] \end{array}$$

where λ represents the coefficient of the gradient penalty item; $P_{\hat{x}}$ generates a straight-line uniform sampling between $P_{g}$ and $P_{r}$ ; $\nabla_{\hat{x}}D\left(\hat{x}\right)$ is the gradient of the discriminator network; ${\left\|\cdot \right\|}_{2}$ stands for second norm of matrix.

### 3.3. Multiple-loop-learning algorithm

**Figure 3:**
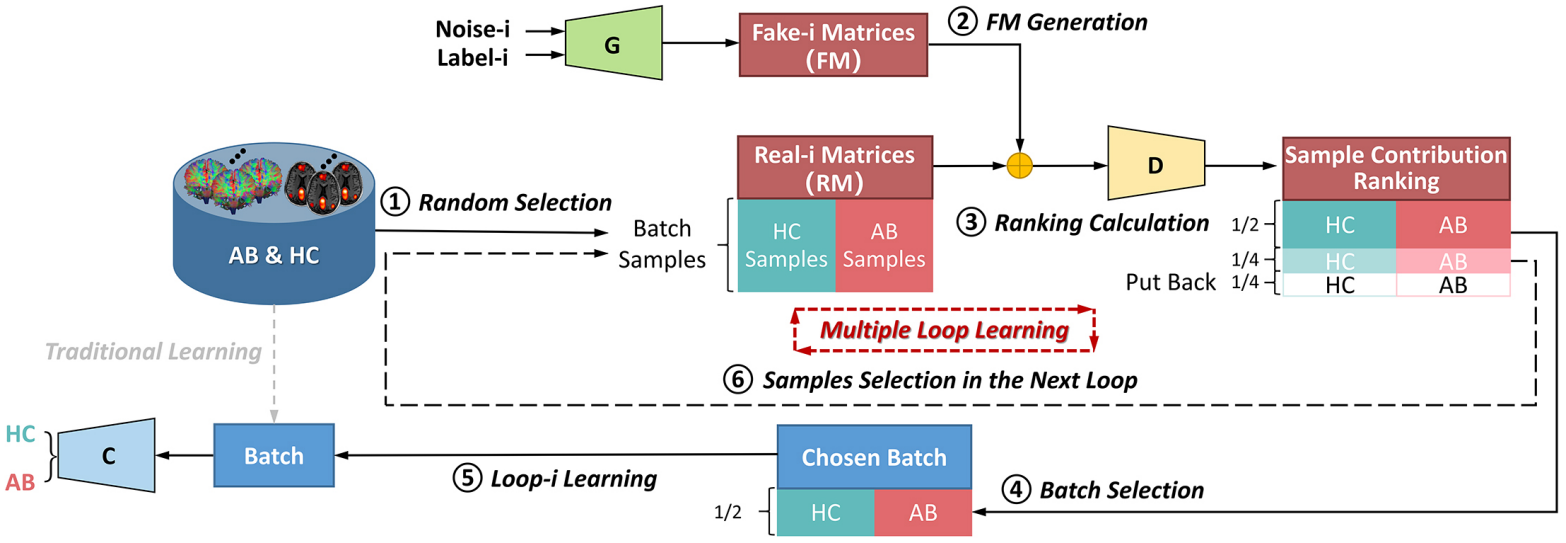
The flowchart of the multiple-loop-learning algorithm with the BNLoop-GAN model. AB, abnormal groups; HC, healthy control groups; C, the classifier; G, the generator; D, the discriminator.

Contrary to most of existing methods that learn data using batch training, this framework splits whole dataset into different subsets for incremental learning. The flowchart of the multiple-loop-learning algorithm in Figure 3 illustrates how to generate incremental training plans.

As shown in Figure 3, in the first loop ( Loop−i,i= 1), the learning process starts by randomly selecting AB and HC samples with a predefined size from the database. Both the Generator and the Discriminator are pre-trained to drive the multiple-loop-learning algorithm. The pre-trained Generator is then employed to generate fake samples that are close to the true distribution of samples. The pre-trained Discriminator is used to process the real and generated samples to compute the sample contribution ranking using Euclidean Distance. To select samples with an easier-to-learn distribution during training loops, the top half of the ranking samples is chosen as the batch for training the classification model, and the middle half to three-quarters of the ranking is prepared for the next loop sample. The bottom quarter of the ranking is put back into the database. Thus far, the first round of loop ends when the classifier has learned a batch. The samples ranked in middle half to three-quarters of the previous round are combined with randomly selected samples to obtain AB and HC samples, which are used in a new round of loop. Multiple-loop-learning is achieved through continuous loop optimization, and the algorithm continues until the classifier converges. Algorithm 1 provides specific details of the algorithm.

**Table:** 

**Algorithm 1:** BNLoop-GAN with multiple-loop-learning.	
**Input:**	the pre-training classifier *C*, generator *G*, and discriminator *D*;
	the brain network matrix of healthy control groups, *M_HC_*;
	the brain network matrix of abnormal groups, *M_AB_*;
	the real matrix, *RM*;
	the fake matrix, *FM*;
	the table of sample contribution ranking of RM, *T_RM_*;
**Output:**	the trained classifier, *C*;
	the accuracy of *C*, *ACC*;
1: Initializing the loop = 0;	
2: Initializing the batch_samples;	
3: while *C* not converge	
4:random select *M_HC_* and *M_AB_* to fill $RM_{\left(M_{HC},M_{AB}\right)}^{loop}$ to batch_samples from the database;	
5:generate $FM_{\left(M_{HC},M_{AB}\right)}^{loop}$ from *G* of random noise;	
6:*T_RM_* ← Euclidean Distance computing *D*( $RM_{\left(M_{HC},M_{AB}\right)}^{loop}$ ) and *D*( $FM_{\left(M_{HC},M_{AB}\right)}^{loop}$ );	
7:choose $RM_{\left(M_{HC},M_{AB}\right)}^{loop}$ of 1/2 top-ranked *T_RM_* for training classifier *C*;	
8:get *ACC*;	
9: $RM_{\left(M_{HC},M_{AB}\right)}^{temp}$ ← $RM_{\left(M_{HC},M_{AB}\right)}^{loop}$ of 1/2–3/4 top-ranked *T_RM_*;	
10: $RM_{\left(M_{HC},M_{AB}\right)}^{loop}$ of 1/4 bottom-ranked *T_RM_* back to the database;	
11:loop + +;	
12:initializing $RM_{\left(M_{HC},M_{AB}\right)}^{loop}$ ;	
13:adding $RM_{\left(M_{HC},M_{AB}\right)}^{temp}$ to $RM_{\left(M_{HC},M_{AB}\right)}^{loop}$ ;	
14: **end while**	
15: **return** *C*, *ACC*	

## 4. Experiments

### 4.1. Datasets and preprocessing

In this paper, the MRI data (including dMRI and rsfMRI) were gathered from the Alzheimer’s Disease Neuroimaging Initiative (ADNI) database.^1^ These subjects were instructed to rest with their eyes open, not to think of anything in particular, and not to fall asleep while collecting rsfMRI. The data set contains 42 AD patients (72.0 ± 17.0, 30F/93M) and 42 gender-age matched HC groups (74.5 ± 10.9, 39F/92M).

To start with, the raw MRI data were converted from DICOM to NIfTI using “dcm2niix” function in the MRIcroGL software.^2^ The bvec and bval files were generated to calculate various diffusion properties on the diffusion gradients and directions. All diffusion-based tractography approaches and subsequent connectome reconstructions were performed in the MRtrix3 software.^3^ Firstly, the initial diffusion images were denoised to increase signal-to-noise ratio. Secondly, gibbs-ringing and bias field correction were performed to reduce artifacts and non-regularities. The eddy current-induced distortion was removed, and head motion error was corrected. Finally, the mean b0 image generated by averaging all the images with b = 0 s/mm^2^ was used to register the diffusion image to the structural MRI using the FSL toolbox.^4^ The rsfMRI data were processed by SPM12 software^5^ with the standard procedures, including slice-timing correction, realignment to the median image, and co-registration to the individual structural MRI.

### 4.2. Brain network computing

#### 4.2.1. Construction of diffusion MRI networks

The constrained spherical deconvolution (CSD) method overcomes the limitations of crossing fibers inherent in the diffusion tensor model (Tournier et al., 2008). Therefore, we performed multi-shell multi-tissue CSD method to obtain the fiber orientation distribution (FOD) (Jeurissen et al., 2014). The white matter pathways of whole brain were reconstructed using probabilistic streamline tractography through the second-order integration over FOD algorithm (Smith et al., 2013). The aparc2009 template (Destrieux et al., 2010) of FreeSurfer was used to divide each brain region, and the connection strength is normalized by the number of streamlines divided by the brain volume, thereby constructing structural brain networks.

#### 4.2.2. Construction of resting-state functional MRI networks

The functional brain networks were constructed using the Nilearn package in Python.^6^ For each subject, the average time series of each brain region were extracted using the aparc2009 template of FreeSurfer. Then, the connectivity characteristics were measured using the Pearson correlation coefficient as shown in Equation 3, by which the matrices of N*N-dimensional functional connectivity were obtained for each subject.

(3) $$r\left(X,Y\right)=\frac{\sum_{i=1}^{N}\left[X\left(i\right)-\bar{X}\right]*\left[Y\left(i\right)-\bar{Y}\right]}{\sqrt{\sum_{i=1}^{N}{\left[X\left(i\right)-\bar{X}\right]}^{2}*\sum_{i=1}^{N}{\left[Y\left(i\right)-\bar{Y}\right]}^{2}}}$$

where r(X,Y) is the Pearson correlation coefficient to measure connected effects between brain regions X and Y ; X(i) and Y(i) represent the time series from two different brain regions respectively, i=1,2,..,N , and N is the number of time points of the subject; $\bar{X}$ and $\bar{Y}$ are the mean values of X(i) and Y(i) respectively.

#### 4.2.3. Joint learning of multi-modal brain networks

Brain disorders exhibit muti-aspect changes in the brain’s structural, functional and dynamic characteristics frequently. The structure forms the foundation of function, while the function is the representation of structure. Multi-modal MRI data analyses can capture complementary characteristics from diverse perspectives, bringing richer information and benefiting classification tasks consequently. We adopted the joint learning method of multi-modal data, that is, superimposing the number of dMRI and rsfMRI brain network channels. The follow-up experimental results can reflect its advantages compared with single-modal brain networks.

#### 4.2.4. Brain network augmentation

**Figure 4:**
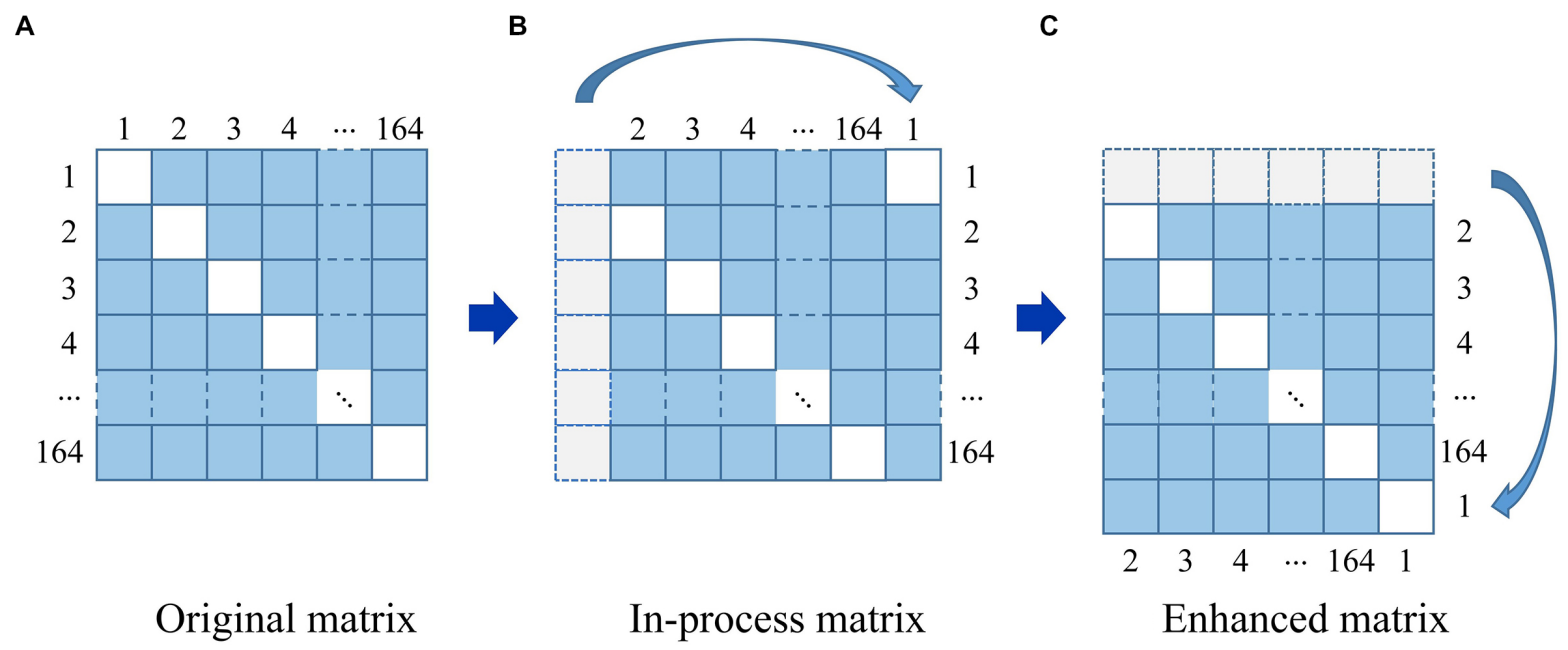
The strategy of data augmentation. **(A)** Original matrix. **(B)** In-process matrix. **(C)** Enhanced matrix.

In order to reduce noise and facilitating normalization of input features, we performed min-max scaling, which involves scaling the data to a range between 0 and 1. Additionally, to prevent over-fitting, the data augmentation techniques were used to expand the training data set, as shown in Figure 4.

For a given image in Figure 4, it can be seen that an original matrix **(A)** is transformed by moving its first column to the last column, generating an in-process matrix **(B)**, and then its first row is moved to the last row, resulting in the enhanced matrix **(C)**. In this way, the strategy of data augmentation will avoid breaking the symmetry of the matrix. We repeat this process on the newly generated enhanced matrix **(C)**, generating 163 additional enhanced matrices from one original brain network matrix corresponding to a single subject, and 13,692 enhanced matrices from 84 subjects in total. We employed all of these matrices, with 80% reserved for training and 20% for testing.

### 4.3. Model description and evaluation indicators

Table 1 presents the architectural parameters of the BNLoop-GAN model in detail. This model is capable of accommodating both single-modal and multi-modal inputs, with the parameter ‘*n*’ in Table 1 denoting the number of modalities.

**Table 1:** The architectural parameters of the BNLoop-GAN model.

Sub-module	Layer	Kernel	Stride	Padding	Size of feature map (height × width × channels)	Activation function
Generator	Input	–	–	–	1 × 100 × *n*	–
	ConvT 1	4	1	0	4 × 4 × 512	ReLU
	ConvT 2	4	2	1	8 × 8 × 256	ReLU
	ConvT 3	4	2	1	16 × 16 × 128	ReLU
	ConvT 4	4	2	1	32 × 32 × 64	ReLU
	ConvT 5	12	1	1	41 × 41 × 32	ReLU
	ConvT 6	4	2	1	82 × 82 × 16	ReLU
	ConvT 7	4	2	1	164 × 164 × *n*	Sigmoid
Discriminator	Input	–	–	–	164 × 164 × *n*	–
	Conv 1	4	2	1	82 × 82 × 16	LeakyReLU
	Conv 2	4	2	1	41 × 41 × 64	LeakyReLU
	Conv 3	10	1	0	32 × 32 × 128	LeakyReLU
	Conv 4	4	2	1	16 × 16 × 256	LeakyReLU
	Conv 5	4	2	1	8 × 8 × 512	LeakyReLU
	Conv 6	4	1	0	5 × 5 × *n*	–
Classifier	Input	–	–	–	164 × 164 × *n*	–
	Conv 7	4	2	1	82 × 82 × 16	LeakyReLU
	Conv 8	4	2	1	41 × 41 × 64	LeakyReLU
	Conv 9	10	1	0	32 × 32 × 128	LeakyReLU
	Conv 10	4	2	1	16 × 16 × 256	LeakyReLU
	Conv 11	4	2	1	8 × 8 × 512	LeakyReLU
	Conv 12	8	2	1	2 × 2 × 1	–

ConvT, Transposed Convolution; Conv, Convolution.

Three indicators are used to evaluate the performance of the model, including accuracy ( ACC ), sensitivity ( SEN ), specificity ( SPE ). The formula is defined as follows:

(4) $$ACC=\frac{TP+TN}{TP+TN+FP+FN}$$

(5) $$SEN=\frac{TP}{TP+FN}$$

(6) $$SPE=\frac{TN}{FP+TN}$$

where FP, FN, TP, and TN denote False Positive, False Negative, True Positive and True Negative assessments, respectively.

Furthermore, due to the complexity of AD diagnosis, the Receiver Operating Characteristic (ROC) curve and Area under the ROC Curve (AUC) are utilized to evaluate the efficacy of binary classification models. The ROC curve plots the true positive rate (*TPR*) against the false positive rate (*FPR*), with *TPR* on the y-axis and *FPR* on the x-axis.

(7) $$TPR=\frac{TP}{TP+FN}$$

(8) $$FPR=\frac{FP}{FP+TN}$$

The AUC is the area under the ROC curve, with values ranging from 0 to 1. A higher AUC indicates better model performance, with 0.5 indicating random guessing and 1 indicating perfect prediction.

## 5. Results and discussions

A case study was performed to examine the classification performance corresponding to single-modal brain networks and multi-modal brain networks, respectively, through the BNLoop-GAN model with the multiple-loop-learning algorithm. In order to evaluate the effectiveness of the different components incorporated into the enhanced-GAN model, we conducted a series of ablation experiments. Table 2 presents the results of these experiments, which were evaluated using three indicators.

**Table 2:** The results of AD classification of different brain network learning strategies using various models.

Model	Brain Network Learning Strategy	*ACC* (%)	*SEN* (%)	*SPE* (%)
FCN	①	77.2	74.7	79.6
	②	75.3	73.9	80.3
	③	81.4	80.2	82.2
FCN with loop of conditional generation	①	77.3	74.5	79.8
	②	75.1	73.6	79.9
	③	81.6	80.5	82.5
FCN with loop of conditional generation and patch-based discrimination	①	78.2	75.1	80.5
	②	76.1	74.2	81.2
	③	82.3	81.0	83.1
FCN with loop of conditional generation and Wasserstein gradient penalty	①	77.8	75.2	80.6
	②	75.9	74.1	81.1
	③	82.1	80.9	83.0
BNLoop-GAN	①	79.1	76.3	81.2
	②	77.0	75.2	81.9
	③	**83.8**	**81.8**	**84.9**

FCN is the fully convolutional neural network. Three brain network learning strategies were utilized, including: ① Single-modal brain networks based on dMRI; ② Single-modal brain networks based on rsfMRI; ③ Multi-modal brain networks based on dMRI and rsfMRI. The bold values represent the highest accuracy, sensitivity and specificity of the BNLoop-GAN model on Multi-modal brain networks based on dMRI and rsfMRI.

Taking into account the necessity of conditional information for driving multiple-loop-learning algorithms, we conducted a series of Loop-based ablation experiments on the baseline FCN model. Compared to the baseline FCN, the FCN with loop of conditional generation demonstrated no significant improvement in indicators for any modality. Simply capturing the similarity of samples through conditional information is not enough to improve loop efficiency. However, FCN with loop of conditional generation and patch-based discrimination, as well as FCN with loop of conditional generation and Wasserstein gradient penalty, both improved performance of the classifier for all modalities. The patch-based discrimination ensures that the generated images have a high degree of similarity to real images in terms of brain regions. The Wasserstein gradient penalty enforces the Lipschitz continuity constraint in the discriminator. Both of them contributes to the improved quality of generated samples. It is worth noting that the BNLoop-GAN model exhibited the highest performance for all three modalities, combining techniques of conditional generation, patch-based discrimination, and Wasserstein gradient penalty to learn the implicit distribution of brain regions. These techniques optimize the model by improving the quality of generated samples, selecting samples with an easier-to-learn distribution during training loops, and providing better performance on a classification task of brain networks. In addition, the evaluation indicators for multi-modal data are higher than those for single-modal data.

**Figure 5:**
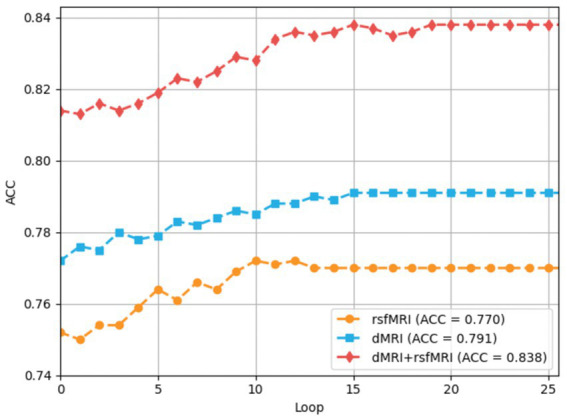
The process of multiple-loop-leaning.

The training processes of each loop driven by the multiple-loop-learning algorithm in the BNLoop-GAN model are shown in Figure 5. The overall trend of the training process reveals that the model’s accuracy can be improved steadily and effectively, regardless of whether single-modal or multi-modal data is used. Furthermore, it can be seen that multi-modal brain networks learned by the BNLoop-GAN model achieve the better accuracy of 83.8% than others related to single-modal brain networks.

**Figure 6:**
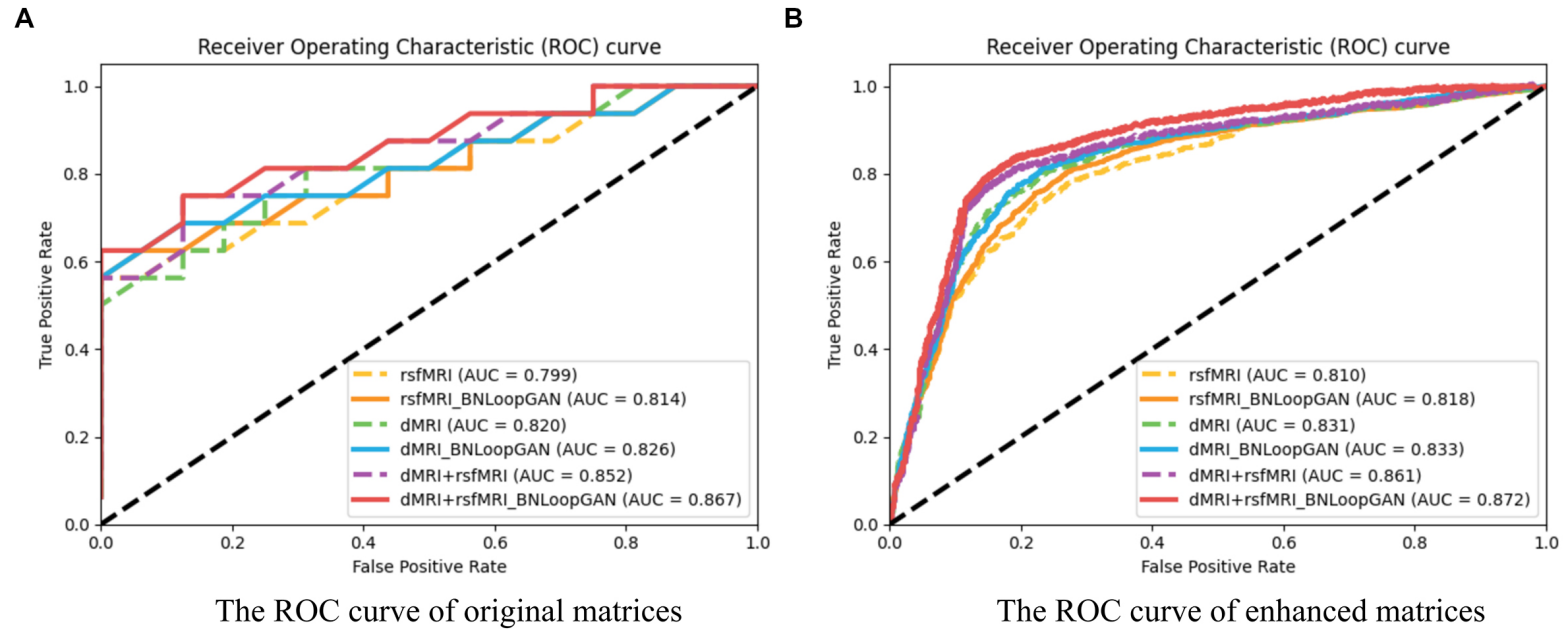
The ROC curves of the BNLoop-GAN model. **(A)** The ROC curve of original matrices. **(B)** The ROC curve of enhanced matrices.

Figure 6 illustrates the computation of ROC curves, which provide a comprehensive representation of performance across different brain network types. Sub-figure **(A)** and **(B)** are constructed using two strategies of original matrices (without brain network augmentation) and enhanced matrices (with brain network augmentation) as a test set to verify the effectiveness of the model. The experimental results prove that data augmentation actually increase the performance of the model. The six curves of each sub-figure are represented by different colors corresponding to different modalities and different model strategies. It can be clearly seen that the classification effect of the model based on multi-modal data is significantly higher than that of single-modal data. Additionally, each dotted ROC curves represent the performance of the basic mode (i.e. FCN), whereas the solid ROC curves depict the performance of the entire BNLoop-GAN model using the multiple-loop-learning algorithm. Compared with the AUC value of the single classifier model, the classification of BNLoop-GAN model has a slight improvement, indicating that the effectiveness of the multiple-loop-learning algorithm can improve the performance of classification, and it performs better in the use of multi-modal data. The AUC value reaches 0.872. All experiments are performed in the same experimental environment with the parameters of the equipment (Intel(R) Core(TM) CPU i7-8750H @ 2.20GHz, 12 CPU cores, 8GB NVIDIA GeForce GTX 1070).

In recent years, an increasing number of studies utilize multiple modalities, such as dMRI and fMRI, to improve the classification of brain networks. Various combination techniques, including feature selection (Yu et al., 2022), data augmentation (Venugopalan et al., 2021), transfer learning (Ghaffari et al., 2022), and more, have been proposed to optimize classification results. For instance, Meng et al. (2022) proposed the multi-modal LassoNet model, which combines fMRI and DTI modalities in a sparse Lasso neural network framework and incorporates connection strength and subject structure to construct a comprehensive multi-modal brain network. The model has achieved a classification accuracy of approximately 90.68% for AD-HC. Mohtasib et al. (2022) conducted a comprehensive connectivity analysis between the default mode network regions using group independent component analysis on rsfMRI data, and examined the paired structural connectivity between the frontal lobe region and the hippocampus using DTI data. They applied both logistic regression and random forest models to classify AD patients and HC groups, achieving an accuracy of 74%. Although some current studies can achieve higher accuracy, it is worth noting that the evaluation strategy is based on k-fold cross-validation (Alorf and Khan, 2022) which is difficult to transfer into real-world scenarios. In this paper, we consider the test samples are not seen in the training phrase. The advantages of the proposed BNLoop-GAN model are as follows. Firstly, an enhanced-GAN model is designed for facilitating to learn the implicit distribution of the brain networks. Secondly, it utilizes the multiple-loop-learning algorithm to select easier-to-learn samples during training loops, continuously improving model classification performance. Lastly, the model can achieve satisfy performance on classification tasks of AD using multi-modal brain network fusion.

## 6. Conclusion

In this paper, the BNLoop-GAN model with a multiple-loop-learning algorithm is proposed to the classification of brain diseases from the brain network perspective. The proposed model is evaluated by the AD classification task, using rsfMRI, dMRI, and their fusion. The experimental results show that the fused brain image learning can achieve a better performance than others, strengthening the importance of fusing structural and functional information. Moreover, the loop learning mode can effectively learn the implicit distribution of brain networks to reduce training complexity and improve classification performance. In the future, more effort will be required to solve the following issues, such as: expanding multi-modal MRI data such as task-state fMRI to capture deeper feature patterns; designing the reasoning rules for representing the main and supplementary modal types with weights and their relations; enriching the “evidence combination-fusion computing” methods for multi-modal brain data.

## Data availability statement

The original contributions presented in the study are included in the article/Supplementary material, further inquiries can be directed to the corresponding authors.

## Author contributions

YC: conceptualization of this study, methodology design and implementation, analysis, interpretation of data, and writing original draft. HK: conceptualization of this study, methodology design and implementation, analysis, and interpretation of data. PL: interpretation of data. J-SP: interpretation of data. NZ: conceptualization of this study, methodology design, interpretation of data, and final approval of the version. JY: conceptualization of this study, interpretation of data, and final approval of the version. All authors contributed to the article and approved the submitted version.

## Conflict of interest

The authors declare that the research was conducted in the absence of any commercial or financial relationships that could be construed as a potential conflict of interest.

## Publisher’s note

All claims expressed in this article are solely those of the authors and do not necessarily represent those of their affiliated organizations, or those of the publisher, the editors and the reviewers. Any product that may be evaluated in this article, or claim that may be made by its manufacturer, is not guaranteed or endorsed by the publisher.

## Footnotes

^1^https://adni.loni.usc.edu

^2^https://www.nitrc.org/projects/mricrogl

^3^https://www.mrtrix.org

^4^https://www.fmrib.ox.ac.uk/fsl

^5^https://www.fil.ion.ucl.ac.uk/spm/software/spm12

^6^https://nilearn.github.io
