# Supplementary material for: BNLoop-GAN: a multi-loop generative adversarial model on brain network learning to classify Alzheimer’s disease
Source: Front Neurosci. 2023 Jun 23;17:1202382. doi: 10.3389/fnins.2023.1202382 (PMC10326383; doi:10.3389/fnins.2023.1202382)
Supplement: Supplementary file 1 [file Data_Sheet_1.PDF]

Supplementary Material

TABLE List of abbreviations used throughout the paper.

Abbreviation	Meaning
AB	abnormal groups
ACC	accuracy
AD	Alzheimer’s Disease
ADNI	the Alzheimer’s Disease Neuroimaging Initiative
AUC	Area under the Receiver Operating Characteristic Curve
BNLoop-GAN	the Loop-based Generative Adversarial Network for Brain Network
cGAN	condition Generative Adversarial Network
CSD	the constrained spherical deconvolution
dMRI	diffusion Magnetic Resonance Imaging
DTI	diffusion tensor imaging
ECFC	evidence combination-fusion computing
FCN	the fully convolutional neural network
fMRI	functional Magnetic Resonance Imaging
FN	false negative
FOD	the fiber orientation distribution
FP	false positive
FPR	the false positive rate
GANs	Generative Adversarial Networks
HC	health control groups
MDMR	multivariate distance matrix regression
MRI	Magnetic Resonance Imaging
ROC	the Receiver Operating Characteristic
rsfMRI	resting-state functional Magnetic Resonance Imaging
SEN	sensitivity

SPE	specificity
TN	true negative
TP	true positive
TPR	the true positive rate
WGAN-GP	Wasserstein Generative Adversarial Network with gradient penalty
